# Health professionals’ experiences and views on obstetric ultrasound in Tanzania: A cross-sectional study

**DOI:** 10.1177/17455057241273675

**Published:** 2024-08-29

**Authors:** Cecilia Bergström, Matilda Ngarina, Muzdalifat Abeid, Hussein Kidanto, Kristina Edvardsson, Sophia Holmlund, Rhonda Small, Jean Paul Semasaka Sengoma, Joseph Ntaganira, Pham Thi Lan, Ingrid Mogren

**Affiliations:** 1 Department of Clinical Sciences, Obstetrics and Gynaecology, Umeå University, Umeå, Sweden; 2 Department of Obstetrics and Gynaecology, Muhimbili University of Health and Allied Sciences, Dar-es-Salaam, Tanzania; 3 Department of Obstetrics and Gynaecology, Muhimbili National Hospital, Dar-es-Salaam, Tanzania; 4 Department of Obstetrics and Gyneacology, Aga Khan University, Dar-es-Salaam, Tanzania; 5 Judith Lumley Centre, School of Nursing and Midwifery, La Trobe University, Melbourne, VIC, Australia; 6 Department of Nursing, Umeå University, Umeå, Sweden; 7 Department of Women’s and Children’s and Reproductive Health, Karolinska Institutet, Stockholm, Sweden; 8 School of Public Health, College of Medicine and Health Sciences, University of Rwanda, Kigali, Rwanda; 9 Department of Dermatology and Venereology, Hanoi Medical University, Hanoi, Vietnam

**Keywords:** obstetric ultrasound, pregnancy, health professionals, clinical management, Tanzania, cross-sectional study, maternal healthcare, ultrasound training

## Abstract

**Background:**

Obstetric ultrasound is considered important for determining gestational age, identifying single or multiple pregnancies, locating the placenta and fetal anomalies and monitoring fetal growth and pregnancy-related complications in order to improve patient management.

**Objectives:**

To explore health professionals’ perspectives on different aspects of obstetric ultrasound in Tanzania regarding self-reported skills in performing ultrasound examinations and what could improve access to and utilization of obstetric ultrasound in the clinical setting.

**Design:**

Cross-sectional study.

**Material and Methods:**

Data was collected between November and December 2017 using a questionnaire based on previous qualitative research results from the CROss Country UltraSound Study (CROCUS Study). Seventeen healthcare facilities in 5 urban and semiurban municipalities in the Dar-es-Salaam region were included, with 636 health professionals participating (physicians, *n* = 307 and midwives/nurses, *n* = 329).

**Results:**

Most health professionals (82% physicians, 81% midwives/nurses) believed that obstetric ultrasound was decisive in the clinical management of pregnancy. Results indicate proficiency gaps across disciplines: 51% of physicians and 48.8% of midwives/nurses reported no or low-level skills in assessing cervical length. Similarly, deficiencies were observed in evaluating the four-chamber view of the fetal heart (physicians: 51%, midwives/nurses: 61%), aorta, pulmonary artery (physicians: 60.5%, midwives/nurses: 65%) and Doppler assessments (umbilical artery: physicians 60.6%, midwives/nurses 56.1%). Compared to midwives/nurses, physicians were significantly more likely to agree or strongly agree that utilization would improve with more ultrasound machines (odds ratio (OR) 2.13; 95% confidence intervals (CI) 1.26–3.61), better quality of ultrasound machines (OR 2.27; 95% CI 1.10–4.69), more training for health professionals currently performing ultrasound (OR 2.11; 95% CI 1.08–4.17) and more physicians trained in ultrasound (OR 2.51; 95% CI 1.30–4.87).

**Conclusions:**

Improving the provision of obstetric ultrasound examinations in Tanzania requires more and better-quality ultrasound machines, enhanced training for health professionals and an increased number of physicians trained in ultrasound use. To further increase the accessibility and utilization of obstetric ultrasound in maternity care in Tanzania, it is essential to provide training for midwives in basic obstetric ultrasound techniques.

## Plain language summary

A study reporting on health professionals’ insights and skills regarding pregnancy scans in Tanzania

This study, conducted in Tanzania, explores health professionals’ perspectives on obstetric ultrasound, an important component believed to increase antenatal care attendance in low-income countries. The research was carried out through a cross-sectional study involving 636 health professionals, primarily physicians and midwives/nurses, to assess their self-reported skills in performing obstetric ultrasound examinations and identify factors that could enhance access to and utilization of obstetric ultrasound in clinical settings. The findings highlight that most health professionals view obstetric ultrasound as crucial in managing pregnancy. However, there is a notable discrepancy in the perceived skills between physicians and midwives/nurses, especially in basic obstetric ultrasound examinations. A majority of health professionals reported either no skills or low-level skills in assessing various aspects like cervical length, fetal heart (four-chamber views), aorta, pulmonary artery and Doppler (umbilical artery). The study suggests that improving access and utilization of obstetric ultrasound could be achieved through increased availability of quality ultrasound machines and enhanced training. Physicians expressed a stronger belief in the positive impact of more ultrasound machines, better machine quality, additional training for current ultrasound examiners and more physicians trained in ultrasound. The data underscores the potential for enhancing obstetric ultrasound provision in Tanzania by investing in better technology, providing more training for healthcare professionals and increasing the number of physicians skilled in ultrasound use. Furthermore, the study recommends targeted training for midwives to enhance their proficiency in basic obstetric ultrasound, ultimately contributing to improved accessibility and utilization of obstetric ultrasound in maternity care in Tanzania.

## Introduction

Antenatal care (ANC) plays an important role in reducing the risk of pregnancy-related complications as well as decreasing maternal and child mortality.^1^ Since 2016, the World Health Organization (WHO) has recommended that all pregnant women should have a minimum of eight contacts with a health provider during pregnancy, as well as one obstetric ultrasound examination before 24 weeks of gestation, to improve maternal, fetal and neonatal outcomes.^2^

Obstetric ultrasound is routinely offered to expectant mothers in high-income countries (HIC). It is primarily used to determine gestational age, single/multiple pregnancy, placental localization and fetal anomaly and later in pregnancy, it is used as a method for surveillance of fetal growth and other pregnancy-related complications.^3,4^ Evidence suggests that obstetric ultrasound may improve patient management^5^ and in low-income countries (LIC) it appears to play an important role in increasing ANC attendance and in motivating pregnant women to deliver at a healthcare facility.^6^ In the last decade, obstetric ultrasound has become more commonly used in LIC^3^ despite barriers such as ultrasound equipment being costly, and the lack of training and insufficient skills of sonographers and physicians.^7,8^ Moreover, the reduction in the size, cost, portability and robustness of ultrasound equipment has undoubtedly increased the interest in the use of obstetric ultrasound to improve pregnancy-related outcomes in LIC.^9–11^

An increase in the utilization of maternal care services has been shown to correspond to a steady decline in maternal mortality in Sub-Saharan African countries.^12^ However, due to the lack of infrastructure, qualified medical personnel and poor access to care, maternal mortality remains high in most LIC.^13^ The quality of ANC in Tanzania remains compromised mostly due to suboptimal care in terms of labour monitoring, poor documentation, infrastructure and compliance, referral delays, treatment, follow-ups and shortage of skilled personnel and medical equipment.^14–18^ Nevertheless, more than 85% of women attended at least four ANC visits during their pregnancy in 2022, an increase of almost 20% compared to 2015–2016.^19^ Despite progress towards the United Nations Millennium Development Goals (MDGs) with a steady decline in maternal mortality rate (MMR) between the years 2000 and 2020, the MMR still stands at 238 per 100,000 live births in the Tanzania.^20^

Accurate data regarding the number of ultrasound machines and trained staff in Tanzania are currently unavailable. However, our prior investigations have underscored significant concerns among Tanzanian healthcare personnel regarding the scarcity of ultrasound machines, the lack of trained personnel to operate them effectively in the clinics and their proficiency in interpreting obstetric ultrasound examinations.^21,22^ Furthermore, our previous research has underscored the pivotal role of obstetric ultrasound in managing complex pregnancies and childbirth in Tanzania.^21,22^

## Aim

Studies on health professionals’ experiences and views on different aspects of obstetric ultrasound are limited in the scientific literature. Thus, the rationale for this study stems from the need to explore health professionals’ perspectives on the pivotal role of obstetric ultrasound in the clinical management of pregnancy alongside accessibility to ultrasound machines. Therefore, we aimed to investigate how health professionals in Tanzania perceive their skills in conducting obstetric ultrasound examinations, identify factors that could enhance the utilization of obstetric ultrasound in clinical settings, and seek to comprehensively understand health professionals’ experiences and viewpoints on obstetric ultrasound in Tanzania’s context. By examining these aspects, we aim to gain insights to inform strategies to optimize obstetric ultrasound utilization and improve maternal and fetal health outcomes in Tanzania. This study is part of the CROss Country UltraSound Study (CROCUS Study) investigating health professionals’ experiences and views of obstetric ultrasound in high-, middle- and low-income countries.^21–33^

## Materials and methods

### Study design

The methodology has been comprehensively outlined in a previous publication from the CROCUS Study.^34^ In essence, the CROCUS Study entailed two phases: (1) a qualitative phase involving focus group discussions with midwives and in-depth interviews with obstetricians, and (2) a quantitative phase featuring a questionnaire derived from the qualitative insights obtained in Phase 1 of CROCUS.^21–32^ In this cross-sectional study, a structured questionnaire was employed to explore various aspects of obstetric ultrasound among obstetricians/gynaecologists/physicians and midwives/nurses involved in antenatal, intrapartum and postpartum care for women in the Dar-es-Salaam region of Tanzania. Purposive selection of health facilities ensured a representative sample of health professionals responsible for the clinical management of pregnant women in the region. The STROBE Guidelines have been used when preparing the manuscript.^35^

### The Tanzanian setting

**Figure 1.**
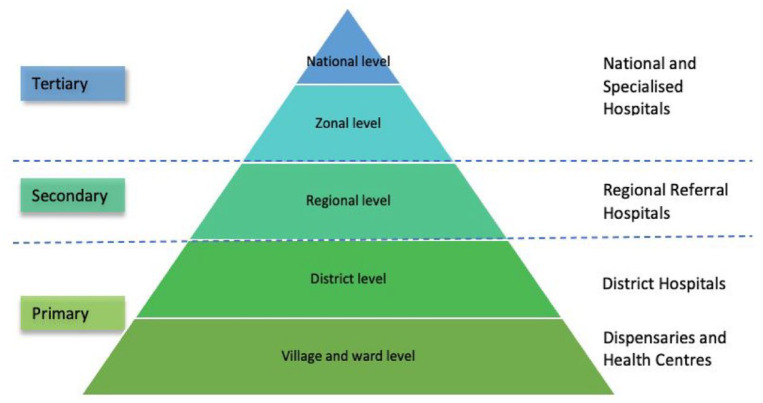
Tanzanian health system pyramid.

Tanzania is located in the eastern part of sub-Saharan Africa within the African Great Lakes Region. Over 100 languages are spoken in Tanzania. However, 90% speak Swahili as a second language, and many educated Tanzanians speak English. Tanzania is a large country of almost 1 million square kilometres, and according to the 2022 census, the Tanzanian population was 62 million.^19^ Life expectancy was 66 years for men^36^ and 68 years for women^37^ in 2021. The Tanzanian health system is decentralized, with health services divided into three levels: national, regional and district levels. The pyramidal referral structure of types of services are as follows (arranged from the bottom and up): Dispensaries and Health Centres, District Hospitals, Regional Referral Hospitals and National and Specialized Hospitals (Figure 1). The number of physicians and midwives/nurses in Tanzania was estimated in 2018 to be 0.1 and 0.6 per 1,000 population, respectively, which is among the lowest rates in the world.^38,39^ Today, there are four government health insurance schemes along with multiple private options. The number of gynaecologists/obstetricians in Tanzania was estimated to be 250 in 2020, according to the Association of Gynaecologists and Obstetricians of Tanzania (personal communication from author MA). A majority of childbirths in Tanzania are assisted by a skilled health provider, with skilled birth attendance rising from 64% in 2015–2016 to 85% in 2022.^19^

### Data collection and validity of data

Four data collectors undertook data collection from November to December 2017, supervised in pairs by authors MN or MA. Authors CB and IM assisted with the data collection during its initial phase. All data collectors were trained by the research team prior to the data collection. The questions and statements in the survey were reviewed to ensure clear understanding. The directors of all study sites in the Dar-es-Salaam region were contacted by authors MN and MA to facilitate appointments for potential study participants to meet with the researchers and data collectors and receive information about the study. Healthcare facilities in five urban and semiurban municipalities in the Dar-es-Salaam region were used to obtain a representative sample of health professionals. The municipal regions included were Ilala, Kinondoni, Ubungo, Temeke and Kigamboni. In total, 17 healthcare facilities were included (1 National Hospital, 1 Zonal hospital (private), 3 Regional Referral Hospitals, 3 District Hospitals (2 private), 1 Poly Clinic (private), 7 Health Centres (1 private), and 1 Dispensary. Participants were recruited at their workplace. Each questionnaire was labelled with a unique identification number and a health facility code. No personal identifying data were collected ensuring the anonymity of the participants. The completed questionnaires were scanned into PDF files to be transferred to Sweden where data were manually entered by an administrator into IBM SPSS Statistics software package. The quality of data entry was evaluated by one author (CB) re-entering every 10th questionnaire and the error rate was calculated to be 1.9%. All original questionnaires are stored in a secure, locked location at Muhimbili National Hospital and all the scanned questionnaires are stored on a USB memory stick in a locked location at Umeå University.

### Study participants

Eligible participants were obstetricians/gynaecologists/physicians and midwives/nurses who worked with obstetric ultrasound examinations or applied results of obstetric ultrasound in clinical practice, in healthcare centres and hospitals in the Dar-es-Salaam region. General physicians were invited to participate in the study since they were responsible for the majority of obstetric ultrasound examinations. Nurses were also invited to participate since they care for a significant portion of women during pregnancy and birth, as the number of midwives in Tanzania is limited. No eligible participants declined participation in the study. In total, 638 health professionals agreed to fill out the questionnaire and return it to the data collector. Two questionnaires were lost in the PDF transfer process, thus the primary sample consisted of 636 participants.

### Independent variables

All questionnaires were dated, and the mean age of participants was calculated as a continuous variable by subtracting the year of birth from 1 January 2017. Gender was dichotomized as female or male. *Current healthcare profession* included the response options: obstetrician/gynaecologist, general practitioner, resident physician, physician other (please specify), midwife, nurse, radiologist/sonographer and other (please specify). The variable current healthcare profession was dichotomized into (1) physicians including obstetrician/gynaecologist, general practitioner, resident physician, radiologist/sonographer and physician other, and (2) midwife/nurse. In addition, one assistant medical officer (AMO) and one clinical officer (CO) participated in the survey and were categorized as physicians. The healthcare facility level was categorized as Dispensaries or Health Centres, District hospitals, Regional Referral Hospital, Zonal Hospitals and National Hospitals (Figure 1) where one Dispensary and one Health Centre were considered faith-based. *Type of healthcare services* was classified as public, private and both public and private.

### Dependent variables

Dependent variables investigating health professionals’ views regarding the role, access, skills and utilization of obstetric ultrasound are presented in Table 1. Variables concerning health professionals’ views on access to obstetric ultrasound and training are presented on a 5-point Likert-point scale with the response options; (1) strongly agree, (2) agree, (3) neutral, (4) disagree, and (5) strongly disagree. Response options for dependent variables concerning self-reported skills for specified obstetric ultrasound examinations were dichotomized into ‘No skills to Low skill level’ and ‘Intermediate to High skill level’. Health professionals’ views regarding dependent variables investigating factors that may improve the utilization of obstetric ultrasound were dichotomized into ‘Not at all or Not very much’ and ‘A fair amount or A great deal’.

**Table 1.** Dependent variables and their response options in the questionnaire.

Statements or questions in the questionnaire	Response options in the questionnaire
How do you rate your skills in relation to the assessment/evaluation of: • Fetal presentation • Localization of placenta • Fetal heart rate • Amount of amniotic fluid • Gestational age; estimated by CRL • Gestational age estimated by biparietal diameter, femur and abdominal diameter • Cervical length • Fetal heart; four-chamber-view • Doppler; umbilical artery	No skills Low skills Intermediate skills High skills
Do you have a role in decision-making regarding clinical management on the basis of obstetric ultrasound?	No Yes, a minor role Yes, a moderate role Yes, a major role
How often do you make decisions based on the results from obstetric ultrasound examinations in your clinical work?	Never On a daily basis On a weekly basis On a monthly basis More rarely than on a monthly basis
What do you believe would help improve the utilization of ultrasound at your clinic/workplace? • More ultrasound machines • Better quality of ultrasound machines • More training for health professionals currently performing ultrasound • More doctors trained in ultrasound • More midwives trained in ultrasound	Not at all Not very much A fair amount A great deal Don’t know
Statements on ultrasound resources and training • Pregnant women in my country have access to dating ultrasound (i.e. estimation of gestational age) • Pregnant women in my country have access to fetal anomaly screening • Pregnant women in my country have access to obstetric ultrasound independent of area of living • Pregnant women in my country have access to obstetric ultrasound independent of income • There are enough resources in my country to provide medically indicated obstetric ultrasound examinations to pregnant women who need it • At my workplace, there is always access to obstetric ultrasound when it is needed • At my workplace, lack of ultrasound training for the ultrasound operator sometimes leads to suboptimal pregnancy management • Maternity care in my country would improve if midwives were qualified to perform basic ultrasound	Strongly agree Agree Neutral Disagree Strongly disagree

CRL: crown-rump-length.

### Questionnaire

The questionnaire was originally developed in English by the research team and included 105 items, that is, statements and questions (Supplemental material). All statements and questions had response options that were either fixed or on a Likert scale. In addition to sociodemographic traits, the survey covered inquiries about access, usage and the significance of obstetric ultrasound in pregnancy surveillance and care. It also included a self-evaluation of proficiency in conducting obstetric ultrasound scans. The questionnaire was translated into Swahili; however, as most educated Tanzanians speak English, it was decided to use the questionnaire originally developed in English. A few adjustments to the original questionnaire were made to better reflect the clinical setting in Tanzania (current profession and current workplace and clarification about how to respond to the questions). To assess understanding and the relevance and appropriateness of the questions and statements, the questionnaire was pilot-tested with one obstetrician/gynaecologist, two resident physicians, two intern physicians, and five midwives, and all found the questions pertinent and interesting.

### Sample size and power

Previous publications regarding the outcomes under study are limited in the scientific literature. Therefore, the calculation of sample size was performed through estimated prevalence based on background characteristics and outcome variables. For the outcome requiring the largest sample size ‘Maternal health interest should always be prioritized over fetal health interest in care’, a sample of 291 obstetricians/physicians and the same number of midwives/nurses (*n* = 291), working in hospital and healthcare centres, was estimated to detect a difference in the proportion of 0.10 with the power of 80% and a significance level of 5%.

### Statistical analysis

Descriptive statistics were used for sociodemographic data where categorical data are presented with proportions and continuous variables are presented with mean values and standard deviation (SD). Pearson chi-square test for categorical data and the independent Student’s *t*-test were used for continuous data when testing for differences between groups. Univariate logistic regression analysis was used to calculate the odds ratio (OR) and 95% confidence intervals (CI). Statistical significance was set at *p* < .05 for all analyses. IBM SPSS Statistics 26 software package was used.

## Results

### Background characteristics of the study sample

**Figure 2.**
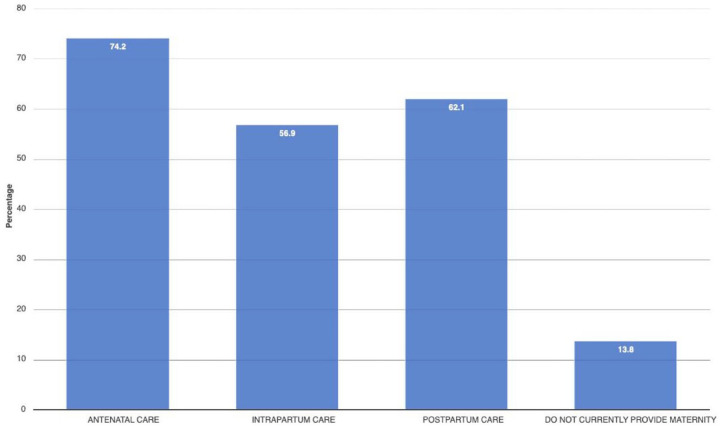
Provision of maternity services.

**Figure 3.**
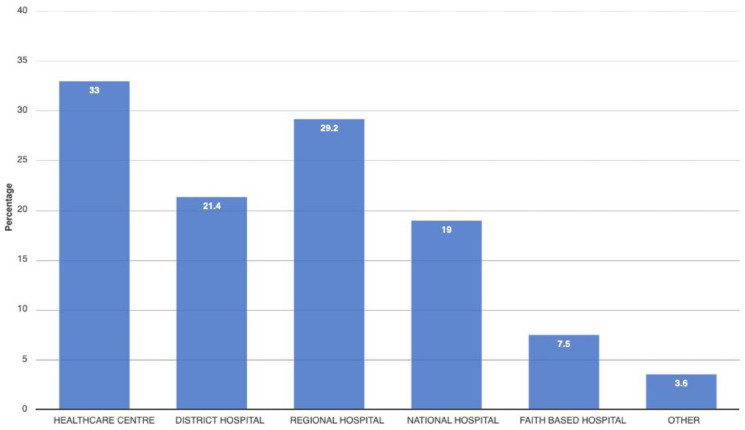
Level of health facility.

A total of 636 questionnaires were included in the analysis with the following: obstetricians/gynaecologist (*n* = 21), general practitioner (*n* = 207), resident physician (*n* = 32), radiologist/sonographer (*n* = 25), physician ‘other’ (*n* = 20), midwife (*n* = 277), nurse (*n* = 52), AMO (*n* = 1) and CO (*n* = 1). The majority of participants were female (64.8%). The vast majority of midwives/nurses were female (85%) and 56.9% of physicians were male. The mean age across gender was 37.3 years (SD 9.1). Most participants were married/cohabiting (70.5%) and more than 75% reported having children. Almost all reported having a religious faith (97.6%). Among participants, mean years in the profession and mean years in healthcare were 10.4 (SD 8.7) and 11.7 (SD 9.3), respectively (Table 2). Most health professionals worked in the public sector and in healthcare centres (72.0%) providing antenatal, intrapartum and postpartum care while 13.8% were not providing maternity care at the time of the survey (Figure 2). Most participants worked in District, Regional, National or Faith-based hospitals (Figure 3) and the vast majority of participants agreed or strongly agreed that obstetric ultrasound examinations are decisive in pregnancy management (82% physicians, 81% midwives/nurses).

**Table 2.** Descriptive information about the study sample and comparisons between physicians and midwives/nurses.

Variable	All health professionals (*N* = 636)	Physicians (*n* = 307)	Midwives/nurses (*n* = 329)	*p-*Value*	
				χ^2^	*F*
Gender	633 (99.5)	306 (99.7)	327 (99.4)		
Male	223 (35.2)	174 (56.9)	49 (15.0)	<.001	
Female	410 (64.8)	132 (43.1)	278 (85.0)		
Age	631 (99.2)	306 (99.7)	325 (98.8)		
Mean; SD	37.3; 9.1	35.7; 8.2	38.8; 9.6		.002
Min–Max	22–69	24–67	22–69		
Marital status	633 (99.2)	307 (100)	326 (99.1)		
Married/cohabiting	433 (68.4)	199 (64.8)	234 (71.8)	<.001	
Cohabiting	13 (2.1)	5 (1.6)	8 (2.5)		
Separated/divorced	16 (2.5)	3 (1.0)	13 (4.0)		
Widowed	16 (2.5)	2 (0.7)	14 (4.3)		
Not married/single	155 (24.5)	98 (31.9)	57 (17.5)		
Children	627 (98.6)	302 (98.4)	325 (98.8)		
Yes	476 (75.9)	217 (71.9)	259 (78.7)	.02	
No	151 (24.1)	85 (28.1)	66 (20.3)		
Do you have a religious faith (any religion)?	609 (95.8)	304 (98.4)	320 (97.3)		
Yes	609 (97.6)	298 (98.0)	311 (97.2)	.72	
No	6 (1.0)	2 (0.7)	4 (1.3)		
I prefer not to answer this question	9 (1.4)	4 (1.3)	5 (1.6)		
Years in profession	626 (98.4)	302 (98.4)	324 (98.5)		
Mean; SD	10.4; 8.7	8.0: 7.6	12.6; 9.1		<.001
Min–Max	0–39	0–36	0–39		
Years in healthcare	630 (99.1)	306 (99.7)	324 (98.5)		
Mean; SD	11.7; 9.3	9.4; 8.2	13.3; 9.8		<.001
Min–Max	0–42	0–36	0–42		
Public/private healthcare	633 (99.5)	305 (99.3)	328 (99.7)		
Public	456 (72.0)	242 (79.3)	214 (65.2)	<.001	
Private	115 (18.2)	40 (13.1)	75 (22.9)		
Both public and private	62 (9.8)	23 (7.5)	39 (11.9)		
Performing ultrasound	627 (98.6)	302 (98.4)	325 (98.8)		
Yes	141 (22.5)	102 (33.8)	39 (12.0)	<.001	
No	486 (77.5)	200 (66.2)	286 (88.0)		
Do midwives perform ultrasounds at your workplace?	611 (96.1)	301 (98.0)	310 (94.2)		
Yes	126 (20.6)	45 (15.0)	81 (26.1)	.001	
No	450 (73.6)	234 (77.7)	216 (69.7)		
Don’t know	35 (5.7)	22 (7.3)	13 (4.2)		
How often do you perform obstetric ultrasound examinations?	141 (22.2)	101 (32.9)	40 (12.2)		
On a daily basis	74 (52.5)	58 (57.4)	16 (40.0)	.30	
On a weekly basis	23 (16.3)	15 (14.9)	8 (20.0)		
On a monthly basis	7 (5.0)	4 (4.0)	3 (7.5)		
More rarely than on a monthly basis	37 (26.2)	24 (23.8)	13 (32.5)		
Those days you perform obstetric ultrasound examinations, estimate the average number of examinations	126 (19.8)	93 (14.6)	33 (0.05)		
Mean; SD	10.9; 10.3	11.3; 10.6	9.7; 9.1		.82
Min–Max	0–75	1–75	0–35		
Do you have a role in decision-making regarding clinical management on the basis of obstetric ultrasound examinations?	631 (99.2)	306 (99.7)	325 (98.8)		
No	127 (20.1)	27 (8.8)	100 (30.8)	<.001	
Yes, a minor role	107 (17.0)	33 (10.8)	74 (22.8)		
Yes, a moderate role	198 (31.4)	83 (27.1)	115 (35.4)		
Yes, a major role	199 (31.5)	163 (53.3)	36 (11.1)		
How often do you make decisions based on the results from obstetric ultrasound examinations in your clinical work?	629 (98.9)	306 (99.7)	323 (98.2)		
Never	111 (17.6)	15 (4.9)	96 (29.7)	<.001	
On a daily basis	385 (61.2)	229 (74.8)	156 (48.3)		
On a weekly basis	35 (5.6)	24 (7.8)	11 (3.4)		
On a monthly basis	13 (2.1)	6 (2.0)	7 (2.2)		
More rarely than on a monthly basis	85 (13.5)	32 (10.5)	53 (16.4)		

Numbers in parentheses are percentages unless otherwise specified.

SD: standard deviation.

* Significance test *p* < .05.

### The role of obstetric ultrasound

Most participants (77.5%), with 88% of midwives/nurses and 66.2% of physicians reported not performing obstetric ultrasound, however, of those who were performing obstetric ultrasound, 57.4% (*n* = 58) of physicians and 40% (*n* = 16) of midwives/nurses performed obstetric ultrasound daily, with a mean number of obstetric ultrasound examinations of 10.9 (SD 10.3) per day (Table 2). Over 50% (*n* = 163) of physicians reported having a major role in decision-making regarding clinical management based on obstetric ultrasound, while 35.4% (*n* = 115) of midwives/nurses reported having a moderate role in clinical management based on obstetric ultrasound. Over 70% of physicians and 48.3% of midwives/nurses reported making decisions on a daily basis in their clinical work based on the results of an obstetric ultrasound (Table 2).

### Access to obstetric ultrasound

The consensus among health professionals is that women in Tanzania have access to dating ultrasound (physicians = 65.7%, midwives/nurses = 75.6%). Physicians were statistically significantly less likely to agree or strongly agree that women in Tanzania have access to fetal anomaly screening compared to midwives/nurses. This agreement was consistent regardless of residential area (physicians = 49.7%, midwives/nurses = 60%) or income (physicians = 44.9%, midwives/nurses = 57.7%) (Table 3).

**Table 3.** Health professionals’ views of access to ultrasound and training analysed by univariate logistic regression using physicians as the reference group.

Variable	Physicians^a^ (*n* = 307)	Midwives/nurses (*n* = 329)	*p-*Value*	OR	CI (95%)	*p-*Value*
			χ^2^			
Pregnant women in my country have access to dating ultrasound (i.e. estimation of gestational age)	306 (99.7)	324 (98.5)				
Strongly agree	79 (25.8)	80 (24.7)	<.001			
Agree	122 (39.9)	165 (50.9)				
Neutral	30 (9.8)	42 (13.0)		2.47	1.60–3.82	<.001
Disagree	61 (19.9	25 (7.7)				
Strongly disagree	14 (4.6)	12 (3.7)				
Pregnant women in my country have access to fetal anomaly screening	304 (99.0)	319 (97.0)				
Strongly agree	38 (12.5)	35 (11.0)	<.001			
Agree	92 (30.3)	145 (45.5)				
Neutral	51 (16.8)	45 (14.1)		1.81	1.28–2.57	<.001
Disagree	75 (24.7)	72 (22.6)				
Strongly disagree	48 (15.8)	22 (6.9)				
Pregnant women in my country have access to obstetric ultrasound independent of area of living	304 (99.0)	323 (98.2)				
Strongly agree	52 (17.1)	55 (17.0)	.006			
Agree	99 (32.6)	139 (43.0)				
Neutral	36 (11.8)	48 (14.9)		1.86	1.30–2.65	<.001
Disagree	81 (26.6)	57 (17.6)				
Strongly disagree	36 (11.8)	24 (7.4)				
Pregnant women in my country have access to obstetric ultrasound independent of income	305 (99.3)	324 (98.5)				
Strongly agree	56 (18.4)	48 (14.8)	<.001			
Agree	79 (25.9)	139 (42.9)				
Neutral	33 (10.8)	49 (15.1)		2.16	1.52–3.05	<.001
Disagree	91 (29.8)	72 (22.2)				
Strongly disagree	46 (15.1)	16 (4.9)				
There are enough resources in my country to provide medically indicated obstetric ultrasound examinations to pregnant women who need it	306 (99.7)	323 (98.2)				
Strongly agree	9 (2.9)	16 (5.0)	<.001			
Agree	29 (9.5)	54 (16.7)				
Neutral	32 (10.5)	61 (18.9)		2.64	1.46–3.51	<.001
Disagree	126 (41.2)	121 (37.5)				
Strongly disagree	110 (35.9)	71 (22.0)				
At my workplace, there is always access to obstetric ultrasound when it is needed	306 (99.7)	322 (97.9)				
Strongly agree	77 (25.2)	74 (23.0)	.97			
Agree	158 (51.6)	173 (53.7)				
Neutral	22 (7.2)	25 (7.8)		1.03	0.67–1.59	.89
Disagree	38 (12.4)	38 (11.8)				
Strongly disagree	11 (3.6)	12 (3.7)				
At my workplace, lack of ultrasound training for the ultrasound operator sometimes leads to suboptimal pregnancy management	305 (99.3)	323 (92.2)				
Strongly agree	79 (25.9)	84 (26.0)	.82			
Agree	137 (44.9)	142 (44.0)				
Neutral	38 (12.5)	35 (10.8)		0.86	0.57–1.30	.48
Disagree	38 (12.5)	42 (13.0)				
Strongly disagree	13 (4.3)	20 (6.2)				
Maternity care in my country would improve if midwives were qualified to perform basic ultrasound examinations	305 (99.3)	325 (98.8)				
Strongly agree	131 (43.0)	156 (48.0)	.05			
Agree	140 (45.9)	123 (37.8)				
Neutral	21 (6.9)	20 (6.2)		0.52	0.30–1.02	.06
Disagree	6 (2.0)	19 (5.8)				
Strongly disagree	7 (2.3)	7 (2.2)				

Numbers in parentheses are percentages unless otherwise specified.

CI: confidence intervals; OR: odds ratio.

a Reference group.

* Significance test *p* < .05.

Nevertheless, approximately 70% of health professionals (physicians = 77.1%, midwives/nurses = 59.5%) disagreed or strongly disagreed that there are enough resources in Tanzania to provide medically indicated obstetric ultrasound examinations to pregnant women (Table 3), with physicians twice as likely to disagree or strongly disagree with the statement compared to midwives/nurses (OR 2.64; 95% CI 1.46–3.51). On the other hand, most health professionals agreed or strongly agreed that there was always access to obstetric ultrasound in their workplace when needed (physicians = 76.8%, midwives/nurses = 76.7%), but the lack of ultrasound training sometimes led to suboptimal pregnancy management (physicians = 70.8%, midwives/nurses = 70%). Nearly 90% of health professionals agreed or strongly agreed that maternity care would improve if midwives were qualified to perform basic obstetric ultrasound examinations.

### Self-rated skills for specified obstetric ultrasound examinations

**Figure 4.**
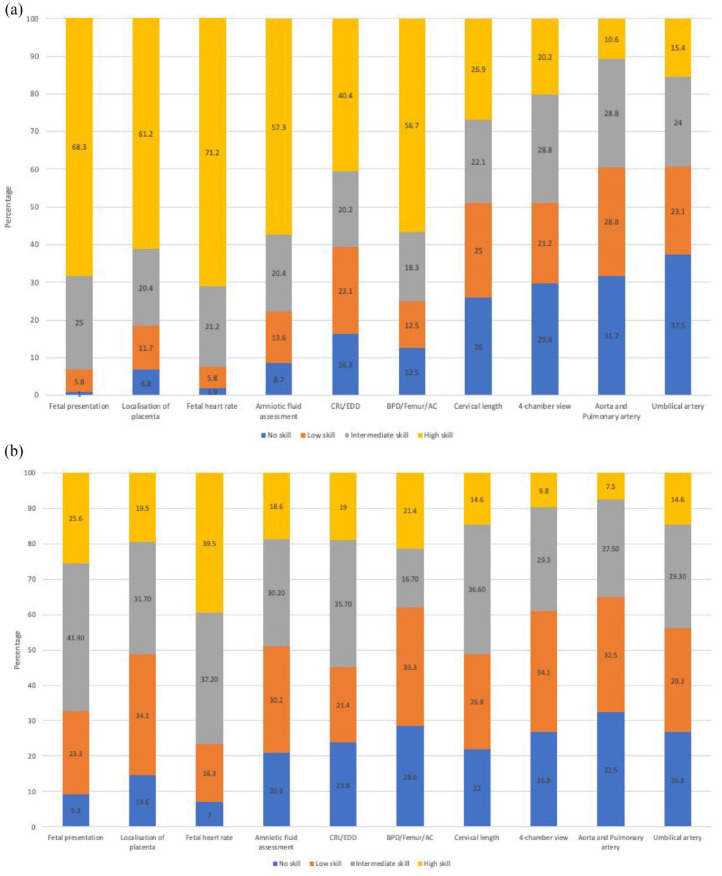
(a) Self-reported ultrasound skills physicians. (b) Self-reported ultrasound skills midwives/nurses.

In Figure 4(a), the data indicate that a majority of physicians performing obstetric ultrasound examinations rated their proficiency as intermediate or high in routine obstetric ultrasound assessments, including assessing fetal presentation (68.3%), placental localization (61.2%), fetal heart rate (71.2%), amniotic fluid (57.3%) and estimating gestational age using biparietal diameter (BPD), femur length (FL) and abdominal circumference (AC) measurements (56.7%). A majority of physicians performing obstetric ultrasound examinations reported no skills to low-level skills regarding the assessment of cervical length (51%), fetal heart: four-chamber view (51%), aorta and pulmonary artery (60.6%) and Doppler: umbilical artery (60.6%) (Table 4). Comparatively, midwives/nurses performing obstetric ultrasound examinations rated their obstetric ultrasound skills lower than physicians across various ultrasound assessments (Figure 4(a) and (b)).

**Table 4.** Self-rated skills for specific obstetric ultrasound examinations.

How do you rate your skills in ultrasound in relation to the assessment/evaluation of	Fetal presentation			Localization of the placenta		
	No to low skill level	Intermediate to high skill level	*p*-Value*	No to low skill level	Intermediate to high skill level	*p*-Value*
Health profession						
Physicians	7 (6.7)	97 (93.3)	<.001	19 (18.4)	84 (81.6)	<.001
Midwives/nurses	14 (32.6)	29 (67.4)		20 (48.8)	21 (51.2)	
Public/private healthcare						
Public	12 (12.0)	88 (88.0)	.36	26 (26.5)	72 (73.5)	.87
Private	5 (16.7)	25 (83.3)		9 (31.0)	20 (69.0)	
Both public and private	4 (25.0)	12 (75.0)		4 (25.0)	12 (75.0)	
Performing ultrasound						
Yes	19 (13.7)	120 (86.3)	.37	35 (25.7)	101 (74.3)	.13
No	2 (25.0)	8 (75.0)		4 (50.0)	4 (50.0)	
How do you rate your skills in ultrasound in relation to the assessment/evaluation of	Fetal heart rate			The assessment/evaluation of amount of amniotic fluid		
	No to low skill level	Intermediate to high skill level	*p*-Value*	No to low skill level	Intermediate to high skill level	*p*-Value*
Health profession						
Physicians	8 (7.7)	96 (92.3)	.009	23 (22.3)	80 (77.7)	<.001
Midwives/nurses	10 (23.3)	33 (76.7)		22 (51.2)	21 (48.8)	
Public/private healthcare						
Public	13 (13.0)	87 (87.0)	.91	31 (31.0)	69 (69.0)	.81
Private	3 (10.0)	27 (90.0)		10 (34.5)	19 (65.5)	
Both public and private	2 (12.5)	14 (87.5)		4 (25.0)	12 (75.0)	
Performing ultrasound						
Yes	17 (12.2)	122 (87.8)	.98	40 (29.0)	98 (71.0)	.046
No	1 (12.5)	7 (87.5)	5 (62.5)	3 (37.5)		
How do you rate your skills in ultrasound in relation to the assessment/evaluation of	Gestational age estimated by CRL			Gestational age estimated by biparietal diameter, femur length and abdominal diameter		
	No to low skill level	Intermediate to high skill level	*p*-Value*	No to low skill level	Intermediate to high skill level	*p*-Value*
Health profession						
Physicians	41 (39.4)	63 (60.6)	.52	26 (25.0)	78 (75.0)	<.001
Midwives/nurses	19 (45.2)	23 (54.8)		26 (61.9)	16 (38.1)	
Public/private healthcare						
Public	43 (43.0)	57 (57.0)	.70	34 (34.0)	66 (66.0)	.19
Private	10 (34.5)	19 (65.5)		14 (46.7)	16 (53.3)	
Both public and private	7 (43.8)	9 (56.3)		3 (20.0)	12 (80.0)	
Performing ultrasound						
Yes	54 (39.1)	84 (60.9)	.045	46 (33.3)	92 (66.7)	.017
No	6 (75.0)	2 (25.0)		6 (75.0)	2 (25.9	
How do you rate your skills in ultrasound in relation to the assessment/evaluation of	Cervical length			Fetal heart: four-chamber view		
	No to low skill level	Intermediate to high skill level	*p*-Value*	No to low skill level	Intermediate to high skill level	*p*-Value*
Health profession						
Physicians	53 (51.0)	51 (49.0)	.81	53 (51.0)	51 (49.0)	.28
Midwives/nurses	20 (48.8)	21 (51.2)		25 (61.0)	16 (39.0)	
Public/private healthcare						
Public	53 (53.0)	47 (47.0)	.35	53 (53.5)	46 (46.5)	.82
Private	11 (37.9)	18 (62.1)		15 (50.0)	15 (50.0)	
Both public and private	8 (53.3)	7 (46.7)		9 (60.0)	6 (40.0)	
Performing ultrasound						
Yes	69 (50.4)	68 (49.6)	.98	70 (51.1)	67 (48.9)	.007
No	4 (50.0)	4 (50.0)		8 (100.0)	-	
How do you rate your skills in ultrasound in relation to the assessment/evaluation of	Fetal heart: aorta and pulmonary artery			Doppler: umbilical artery		
	No to low skill level	Intermediate to high skill level	*p*-Value*	No to low skill level	Intermediate to high skill level	*p*-Value*
Health profession						
Physicians	63 (60.6)	41 (39.4)	.63	63 (60.6)	41 (39.4)	.62
Midwives/nurses	26 (65.0)	14 (35.0)		23 (56.1)	18 (43.9)	
Public/private healthcare						
Public	60 (60.6)	39 (39.4)	.60	57 (57.6)	42 (42.2)	.49
Private		17 (58.6)	12 (41.4)	17 (56.7)	13 (43.3)	
Both public and private		11 (73.3)	4 (26.7)	11 (73.3)	4 (26.7)	
Performing ultrasound						
Yes	82 (60.3)	54 (39.7)	.12	80 (58.4)	57 (41.6)	.35
No	7 (87.5)	1 (12.5)		6 (75.0)	2 (25.0)	

Numbers in parentheses are percentages unless otherwise specified.

CRL: crown-rump-length.

* Significance test *p* < .05.

### Improving utilization of obstetric ultrasound

**Figure 5.**
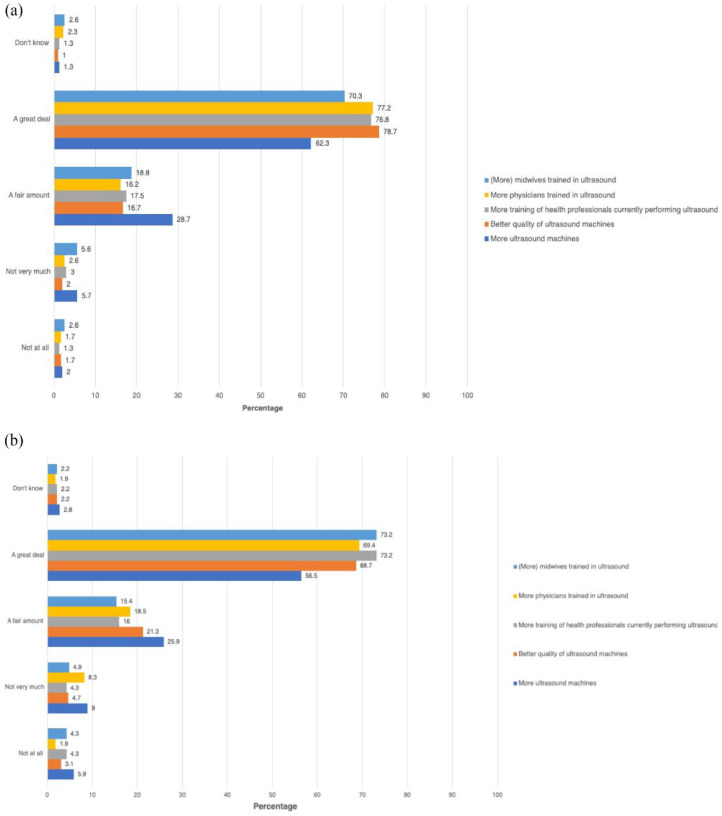
(a) Factors that may improve the utilization of obstetric ultrasound physicians. (b) Factors that may improve the utilization of obstetric ultrasound midwives/nurses.

Approximately 9 of 10 health professionals reported that utilization of obstetric ultrasound would improve ‘A fair amount or A great deal’ if there were more ultrasound machines (physicians = 92.2%, midwives/nurses = 84.8% ) and better-quality machines (physicians = 96.3%, midwives/nurses = 92.0% ), more training for health professionals currently performing obstetric ultrasound (physicians = 95.6%, midwives/nurses = 91.2% ), and if there were more physicians (physicians = 95.6%, midwives/nurses = 89.6%) and midwives (physicians = 91.5%, midwives/nurses = 90.6%) trained in obstetric ultrasound (Table 5). The distribution of answers is shown in more detail in Figure 5(a) and (b). The numbers were similar for health professionals working in the public or private sector or both, as well as for those performing or not performing obstetric ultrasound (Table 5). Compared to midwives/nurses, physicians were more likely to agree or strongly agree that utilization would improve with more ultrasound machines (OR 2.13; 95% CI 1.26–3.61), better quality of ultrasound machines (OR 2.27; 95% CI 1.10–4.69), more training for health professionals currently performing ultrasound (OR 2.11; 95% CI 1.08–4.17) and more physicians trained in ultrasound (OR 2.51; 95% CI 1.30–4.87). Compared to health professionals working in the public healthcare sector, health professionals working in private healthcare were significantly less likely to agree or strongly agree that more ultrasound machines (OR 0.35; 95% CI 0.20–0.62), better quality of ultrasound machines (OR 0.32; 95% CI 0.16–0.66), more training for health professionals currently performing ultrasound (OR 0.52; 95% CI 0.24–1.09) and more training of physicians (OR 0.28; 95% CI 0.15–0.55) and midwives/nurses (OR 0.45; 95% CI 0.24–0.83) would improve utilization of obstetric ultrasound (Table 5).

**Table 5.** Factors that may improve utilization of obstetric ultrasound analysed by univariate logistic regression.

Variable	More ultrasound machines						Better quality of ultrasound machines					
	Not at all or not very much	A fair amount or a great deal	*p*-Value*	OR	95% CI	*p*-Value*	Not at all or not very much	A fair amount or a great deal	*p*-Value*	OR	95% CI	*p*-Value*
Health profession												
Midwives/nurses	48 (15.2)	267 (84.8)		1			25 (8.0)	287 (92.0)		1		
Physicians	23 (7.8)	273 (92.2)	.004	2.13	1.26–3.61	.005	11 (3.7)	286 (96.3)	.02	2.27	1.10–4.69	.03
Public/private healthcare												
Private	23 (21.5)	84 (78.5)		1			14 (13.0)	94 (87.0)		1		
Public	39 (8.8)	406 (91.2)	<.001	0.35	0.20–0.62	<.001	20 (4.6)	419 (95.4)	.003	0.32	0.16–0.66	.002
Performing ultrasound												
No	49 (10.1)	416 (89.5)		1			24 (5.2)	439 (94.8)		1		
Yes	21 (15.3)	116 (84.7)	.12	0.65	0.38–1.13	.13	12 (8.8)	125 (91.2)	.12	0.57	0.28–1.71	.13
	More training for health professionals currently performing ultrasound						More physicians trained in ultrasound					
	Not at all or not very much	A fair amount or a great deal	*p*-Value*	OR	95% CI	*p*-Value*	Not at all or not very much	A fair amount or a great deal	*p*-Value*	OR	95% CI	*p*-Value*
Health profession												
Midwives/nurses	28 (8.8)	290 (91.2)		1			33 (10.4)	285 (89.6)		1		
Physicians	13 (4.4)	285 (95.6)	.03	2.11	1.08–4.17	.03	13 (4.4)	282 (95.6)	.005	2.51	1.30–4.87	.006
Public/private health care												
Private	11 (10.0)	99 (90.0)		1			18 (16.4)	92 (83.6)		1		
Public	24 (5.4)	419 (94.6)	.13	0.52	0.24–1.09	.08	23 (5.2)	416 (94.8)	<.001	0.28	0.15–0.55	<.001
Performing ultrasound												
No	29 (6.1)	444 (93.9)		1			34 (7.3)	433 (92.7)		1		
Yes	11 (8.1)	125 (91.9)	.42	0.74	0.36–1.53	.42	12 (8.8)	125 (91.2)	.57	0.82	0.41–1.63	.57
	(More) midwives trained in ultrasound											
	Not at all or not very much	A fair amount or a great deal	*p*-Value*	OR	95% CI	*p*-Value*						
Health profession												
Midwives/nurses	30 (9.4)	288 (90.6)		1								
Physicians	25 (8.5)	270 (91.5)	.68	1.13	0.65–1.96	.68						
Public/private healthcare												
Private	17 (15.7)	91 (84.3)		1								
Public	34 (7.7)	408 (92.3)	.03	0.45	0.24–0.83	.01						
Performing ultrasound												
No	42 (9.0)	426 (91.0)		1								
Yes	13 (9.6)	123 (90.4)	.84	0.93	0.49–1.79	.84						

Numbers in parentheses are percentages unless otherwise specified.

CI: confidence intervals; OR: odds ratio.

* Significance test *p* < .05.

## Discussion

This study aimed to comprehensively explore health professionals’ perspectives on different aspects of obstetric ultrasound examinations in Tanzania. Most health professionals in our study believed that obstetric ultrasound is decisive in the management of pregnancy and that inadequate training in obstetric ultrasound sometimes leads to suboptimal management, a finding congruent with the results from other countries included in the CROCUS Study.^21–33^

Reducing MMR remains one of the top priorities of the MDGs, with concerted efforts contributing to the decline in MMR in Sub-Saharan countries.^12^ However, many LIC continue to demonstrate unacceptably high MMRs due to infrastructure deficiencies, inadequate access to care, and limited screening for early obstetric risks.^13^ Despite a demonstrated decline in MMR in Tanzania, the current MMR remains high in comparison to other LIC.^20^ It has been suggested that a large number of maternal deaths are caused by preventable conditions.^40–43^ Despite more than 85% of women in Tanzania attending at least four ANC visits during pregnancy,^19^ it is still just half of the recommended ANC contacts now advocated by the WHO,^2^ indicating a need for enhanced screening for prevention, early detection and treatment during pregnancy.^44^ Increased availability and use of obstetric ultrasound as part of ANC in LIC^3^ may lead to an increase in ANC utilization, improved safety of pregnancy and delivery, more hospital deliveries and thus a reduction of the MMR.^6,45–47^ Nevertheless, only 20% of the health professionals in our study working in obstetric care reported performing obstetric ultrasound examinations themselves.

The use of portable and more robust ultrasound equipment is becoming increasingly more common^48^ and may offer better access to obstetric ultrasound for women in rural areas. This technology can help with triaging emergencies, decrease treatment delays and facilitate timely referrals, without compromising on quality,^48^ thereby improving pregnancy-related outcomes.^9–11,49,50^ Health professionals in this study expressed significant concerns about the lack of ultrasound equipment and resources to provide medically indicated obstetric ultrasound examinations, which aligns with the findings in the qualitative CROCUS Study in Tanzania.^22^ Interestingly, these concerns stand in contrast to the majority of health professionals agreeing or strongly agreeing that there was always access to obstetric ultrasound when needed and that women in Tanzania have access to obstetric ultrasound regardless of income and residential area. This discrepancy has been noted in a previous publication based on the CROCUS Study in Rwanda.^33^ The results revealed that although health professionals generally recognize the importance and necessity of obstetric ultrasound, many report insufficient resources and equipment to adequately meet these needs. However, within their specific workplaces, access was generally perceived as available when needed, suggesting a disparity between local and broader resource availability perceptions. This highlights a complex interplay between perceived local accessibility and broader systemic resource limitations.

The self-reported levels of obstetric ultrasound skills in our study provide insight into participants’ ultrasound competence, which indirectly reflects their level of education and training in obstetric ultrasound.^33^ Based on the findings of our study, it is evident that there is a variation of self-reported skills in obstetric ultrasound examinations among health professionals. While most participants perceived themselves to have intermediate to high skills in routine obstetric ultrasound examinations, such as assessing fetal presentation, placental localization, fetal heart rate, amniotic fluid and determining gestational age using BPD, FL and AC, they reported having no skills or low-level skills in more advanced obstetric ultrasound examinations, including the assessment of fetal heart: four-chamber view, aorta, pulmonary and umbilical artery, a finding congruent with another study.^51^ The variation in skill levels raises important considerations regarding the training and education of health professionals in obstetric ultrasound. This is particularly important given that over 60% of the participants reported making daily clinical decisions based on obstetric ultrasound examinations, and a similar percentage indicated having a moderate to major role in decision-making regarding the clinical management of pregnancy based on obstetric ultrasound findings. Previous findings from the qualitative part of the CROCUS Study in Tanzania indicated that there was a need for further training of physicians in how to operate ultrasound machines as well as interpretation of images to increase access and adequate utilization of obstetric ultrasound.^22^ In this larger study, insufficient or lack of training was considered to lead to suboptimal management in pregnancy by most health professionals. Previous research has demonstrated that healthcare workers can be effectively trained in basic obstetric ultrasound use and that skills are retained over time.^8,47,52^ In addition, healthcare workers trained in obstetric ultrasound feel more empowered and motivated to screen pregnant women for obstetric risks.^47^ The variation in proficiency levels in obstetric ultrasound examinations in our study highlights the necessity for structured, standardized theoretical and hands-on training programmes aimed at enhancing skills in both routine and advanced obstetric ultrasound for physicians and midwives alike in order to improve maternal outcomes.^53–55^

Despite the important role of obstetric ultrasound in pregnancy and childbirth, understaffed and underfinanced health systems, with the addition of an ineffective referral system and inadequate infrastructure for obstetric emergencies, will continue to struggle with lack of capacity.^56^ Yet it is important to understand the potential positive impact of increased availability and utilization of obstetric ultrasound together with sufficiently trained health professionals in LIC. Miller et al^44^ coined the terms ‘too little, too late’ (TLTL) and ‘too much, too soon’ (TMTS) where TLTL indicates inadequate resources or withheld or unavailable care until too late, thus associated with high MMR. In contrast, TMTS implies over-medicalization of normal pregnancy and birth, that is, unnecessary use of non-evidence-based interventions. TLTL is mostly ascribed to LIC and TMTS to HIC; however, these two extremes can coexist in the same country. Participants in this study reported that improved quality of ultrasound equipment, more training opportunities and increased ultrasound machines, along with training more midwives to perform obstetric ultrasound, would improve the clinical management of pregnant women in Tanzania. These improvements may also represent a vital component in a broader strategy to enhance pregnancy-related outcomes and to reduce MMR.^57^ However, to achieve substantial and lasting improvements, the entire healthcare system must undergo reforms to establish a safe, high-quality, and evidence-based obstetric and neonatal care chain of care LIC.^56^

### Limitations

Previous studies addressing health professionals’ views of obstetric ultrasound are lacking, thus our sample size and power calculation were based on the assumption of proportions for outcome variables in relation to background variables. A total of 636 participants (*n* = 307 physicians and *n* = 329 midwives/nurses) were recruited, enabling sufficient power for all pre-planned subgroup analyses to be undertaken. We aimed to recruit a representative sample of health professionals, including both ultrasound operators and non-operators from public and private healthcare, different-level hospitals and healthcare centres. However, by limiting recruitment to the urban and semiurban Dar-es-Salaam region, we cannot be certain that our sample is representative of the whole of Tanzania. Other limitations of the study include the potential for data obsolescence, given that the data was collected in 2017. Since then, practices and challenges in obstetric ultrasound provision in Tanzania may have evolved, impacting the relevance of the findings to current circumstances.

The questionnaire was translated into Swahili, but in the end, only the English version was used (with a few adjustments). Despite pilot testing of the English version and that most educated Tanzanians speak English, it is possible that some questions were not fully understood by all participants. It is noteworthy, however, that none of the invited participants declined to take part in this study, and there were few missing responses. This suggests that the questionnaire was both relevant and well-received by the participants. The high participation rate and the completeness of the responses indicate that the questions were generally clear and comprehensible and that the participants were engaged and found the content of the questionnaire pertinent to their experiences.

The self-reported nature of obstetric ultrasound examination skill levels raises concerns about the validity of these responses. Participants’ ability to distinguish between low and intermediate levels of proficiency can be inherently subjective and ambiguous. This ambiguity underscores the necessity for further investigation into the assessment methods used and highlights the need for more objective measures of proficiency to ensure accurate and reliable evaluations of skill levels. Implementing ultrasound simulation and providing sufficient training in obstetric ultrasound have been demonstrated to improve skills and competency, offering a more standardized approach to evaluating and enhancing proficiency among healthcare professionals.^58^

## Conclusion

Most participating health professionals in Tanzania stated that obstetric ultrasound is decisive in the clinical management of pregnancy and that access to and utilization of obstetric ultrasound in Tanzania would improve with more and better-quality ultrasound machines. Yet, most health professionals reported inadequate skill levels regarding more advanced obstetric ultrasound examinations. In addition to more adequate resources to increase access to and utilization of obstetric ultrasound to improve the provision of obstetric care in Tanzania, it is evident that more training of health professionals is also required. To further improve maternity care in Tanzania, there was also strong support for more midwives to be trained in basic obstetric ultrasound examinations to increase access to and utilization of obstetric ultrasound.

## Supplemental Material

sj-docx-1-whe-10.1177_17455057241273675 – Supplemental material for Health professionals’ experiences and views on obstetric ultrasound in Tanzania: A cross-sectional study Supplemental material, sj-docx-1-whe-10.1177_17455057241273675 for Health professionals’ experiences and views on obstetric ultrasound in Tanzania: A cross-sectional study by Cecilia Bergström, Matilda Ngarina, Muzdalifat Abeid, Hussein Kidanto, Kristina Edvardsson, Sophia Holmlund, Rhonda Small, Jean Paul Semasaka Sengoma, Joseph Ntaganira, Pham Thi Lan and Ingrid Mogren in Women’s Health

## Declarations

**Ethical approval and consent to participate:** Ethical approval for this study was obtained from the National Institute for Medical Research and the Ministry of Health, Community Development, Gender, Elderly & Children, Tanzania (Reference: NIMR/HQ/R.8a/Vol. IX/2583). Participation in the study was voluntary and all participants received verbal and written information and signed a consent form prior to filling out the questionnaire.

**Consent for publication:** None.

**Availability of data and materials:** The datasets used and/or analysed during the current study are available from the corresponding author upon reasonable request.
