# Supplementary material for: Health professionals’ experiences and views on obstetric ultrasound in Tanzania: A cross-sectional study
Source: Womens Health (Lond). 2024 Aug 29;20:17455057241273675. doi: 10.1177/17455057241273675 (PMC11363060; doi:10.1177/17455057241273675)
Supplement: sj-docx-1-whe-10.1177_17455057241273675 – Supplemental material for Health professionals’ experiences and views on obstetric ultrasound in Tanzania: A cross-sectional study [file sj-docx-1-whe-10.1177_17455057241273675.docx]

**Data Collector:**


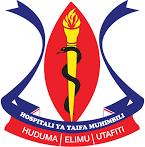

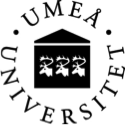


**Number Questionnaire:**

**Date:**

**Health Facility Code:**

The CROss Country Ultrasound Study – CROCUS

Thank you for participating in this study. Below you will find questions related to the use of ultrasound in pregnancy, and also some other aspects of maternity care. This study is being undertaken in six low-, middle-, and high-income countries, with the same questionnaire used across countries. You may find that some questions are less relevant to your setting. However, please read each question carefully and choose the most suitable response option/s.

The confidentiality of all participants will be protected. You are *not* being asked to provide any identifying information. Please mark with an **X** in the box for each of the questions.

| **1.** | What year were you born? |  |
| --- | --- | --- |
| **2.** | Sex: | - Female - Male |
| **3.** | Do you have a religious faith? (any religion) | - Yes - No - I prefer not to answer this question |
| **4.** | What is your marital status? | □ Married |
|  |  | □ Cohabiting |
|  |  | □ Separated/Divorced |
|  |  | □ Widowed |
|  |  | □ Not married/Single |
| **5.** | Do you have children? | □ Yes |
|  |  | □ No |
| **6.** | What is your current profession? | - Obstetrician/gynaecologist - General practitioner - Resident physician   □ Physician, other, please specify.......................................................   - Midwife - Nurse |
|  |  | □ Radiologist/sonographer  □ Other (please specify)...................................................................... |
| **7.** | How many years have you been working in your profession? |  |
|  |  | Number of years: |
| **8.** | How many years have you been working in medical/health care in  total? |  |
|  |  | Number of years: |

| 1. Current workplace: □ Health centre   (Please tick all that apply) □ District hospital   - - Regional hospital   - National hospital   - Faith based hospital   - Other type of health facility, please specify:.............................................................................................  1. Do you work in public or private □ Public health care? □ Private    - Both public and private   □   1. Which of the following maternity □ Antenatal services do you provide? □ Intrapartum   (Please tick all that apply) □ Postpartum   - - I do not currently provide maternity care | | |
| --- | --- | --- |
| **12.** | Do you yourself perform obstetric ultrasound examinations in your clinical work? | - Yes - No   If **No**, go to question **16.** |
| **13.** | How often do you perform obstetric ultrasound examinations? (Please choose **one** alternative that reflect your situation best) | - On a daily basis - On a weekly basis - On a monthly basis - More rarely than on a monthly basis |
| **14.** | Those days you perform obstetric ultrasound examinations, estimate the average number of  examinations |  |
|  |  | Number of examinations: |

**15.** How do you rate your skills in ultrasound in relation to the assessment/evaluation of:

|  | | **No skills** |  | ***Skill level*** |  |
| --- | --- | --- | --- | --- | --- |
|  |  |  | **Low** | **Inter-**  **mediate** | **High** |
| a | Fetal presentation | □ | □ | □ | □ |
| b | Localisation of the placenta | □ | □ | □ | □ |
| c | Fetal heart rate | □ | □ | □ | □ |
| d | Amount of amniotic fluid | □ | □ | □ | □ |
| e | Gestational age estimated by CRL (crown- rump-length) | □ | □ | □ | □ |
| f | Gestational age estimated by biparietal diameter, femur length and abdominal  diameter | □ | □ | □ | □ |
| g | Cervical length | □ | □ | □ | □ |
| h | Fetal heart: 4 chamber view | □ | □ | □ | □ |
| i | Fetal heart: aorta and pulmonary artery | □ | □ | □ | □ |
| j | Doppler: umbilical artery | □ | □ | □ | □ |

| **16.** | Do you have a role in decision-  making regarding clinical management on the basis of obstetric ultrasound examinations? | - No - Yes, a minor role - Yes, a moderate role - Yes, a major role |
| --- | --- | --- |
| **17.** | How often do you make decisions based on the results from obstetric ultrasound examinations in your clinical work? | - Never - On a daily basis - On a weekly basis - On a monthly basis - More rarely than on a monthly basis |

1. What do **you** believe would help improve the utilisation of ultrasound at your clinic/work place?

|  |  | **Not at all** | **Not very**  **much** | **A fair**  **amount** | **A great**  **deal** | **Don’t**  **know** |
| --- | --- | --- | --- | --- | --- | --- |
| a | More ultrasound machines | □ | □ | □ | □ | □ |
| b | Better quality of ultrasound machines | □ | □ | □ | □ | □ |
| c | More training for health professionals currently  performing ultrasound | □ | □ | □ | □ | □ |
| d | More doctors trained in ultrasound | □ | □ | □ | □ | □ |
| e | (More) midwives trained in ultrasound | □ | □ | □ | □ | □ |

1. Are there ***any*** guidelines at your clinic/work place for use of ultrasound in pregnancy **from the second trimester**?

|  |  | **Yes** | **No** | **Don’t know** |
| --- | --- | --- | --- | --- |
| a | Clinic guidelines? | □ | □ | □ |
| b | Regional guidelines? | □ | □ | □ |
| c | National guidelines? | □ | □ | □ |

**If a, b and c = No, go to question21.**

1. From your own experience, to what extent are these guidelines followed?

|  |  | **Don’t know** | **Not at all** | **To a small extent** | **To a moderate**  **extent** | **To a great extent** |
| --- | --- | --- | --- | --- | --- | --- |
| a | Clinic guidelines? | □ | □ | □ | □ | □ |
| b | Regional guidelines? | □ | □ | □ | □ | □ |
| c | National guidelines? | □ | □ | □ | □ | □ |

Statements below represent views obtained through qualitative interviews with maternity care professionals in six low-, medium- and high-resource countries. You may agree or disagree with, or feel neutral about the statements. For each statement, please state your level of agreement.

1. **Ultrasound resources and training**

| Please circle **the number** which best indicates your views about each statement | | **Strongly agree** | **Agree** | **Neutral** | **Disagree** | **Strongly disagree** |
| --- | --- | --- | --- | --- | --- | --- |
| a | Pregnant women in my country have access to **dating ultrasound** (i.e. estimation of gestational age) | **1** | **2** | **3** | **4** | **5** |
| b | Pregnant women in my country have access to **fetal anomaly screening** | **1** | **2** | **3** | **4** | **5** |
| c | Pregnant women in my country have access to obstetric ultrasound independent of area of living | **1** | **2** | **3** | **4** | **5** |
| d | Pregnant women in my country have access to obstetric ultrasound independent of income | **1** | **2** | **3** | **4** | **5** |
| e | There are enough resources in my country to provide **medically indicated** obstetric ultrasound examinations to pregnant women who need it | **1** | **2** | **3** | **4** | **5** |
| f | At my workplace, there is always access to obstetric ultrasound when it is needed | **1** | **2** | **3** | **4** | **5** |
| g | At my workplace, lack of ultrasound training of the ultrasound operator sometimes leads to suboptimal pregnancy management | **1** | **2** | **3** | **4** | **5** |
| h | Maternity care in my country would improve if midwives were qualified to perform basic ultrasound examinations | **1** | **2** | **3** | **4** | **5** |
| i | Obstetric ultrasound examinations are often performed for non-medical purposes in my country | **1** | **2** | **3** | **4** | **5** |
| j | Commercialisation of ultrasound is a problem in my hospital/clinic | **1** | **2** | **3** | **4** | **5** |
| k | Commercialisation of ultrasound is a problem in my country | **1** | **2** | **3** | **4** | **5** |
| l | Pregnant women should be able to have non-medical ultrasounds on their own request | **1** | **2** | **3** | **4** | **5** |
| m | Do you feel that pregnant women expect to have an ultrasound during consultations, even when there is no medical indication for ultrasound? | **1** | **2** | **3** | **4** | **5** |

1. **The role of ultrasound in clinical management of pregnancy**

| Please circle **the number** which best indicates your views about each statement | | **Strongly agree** | **Agree** | **Neutral** | **Disagree** | **Strongly disagree** |
| --- | --- | --- | --- | --- | --- | --- |
| a | Ultrasound is decisive in pregnancy management | **1** | **2** | **3** | **4** | **5** |
| b | Every woman should undergo ultrasound examination in pregnancy to determine gestational age | **1** | **2** | **3** | **4** | **5** |
| c | It is irresponsible of a pregnant woman to  decline a dating scan | **1** | **2** | **3** | **4** | **5** |
| d | Ultrasound is safe to use for the pregnant woman and the fetus irrespective of the number of examinations | **1** | **2** | **3** | **4** | **5** |
| e | Ultrasound is important for expectant parents  to bond with their fetus during pregnancy | **1** | **2** | **3** | **4** | **5** |

1. **Information in relation to ultrasound examinations**

| Please circle **the number** which best indicates your views about each statement | | **Not relevant in my clinic** | **Not relevant in my country** | **Strongly agree** | **Agree** | **Neutral** | **Disagree** | **Strongly disagree** | **Don’t**  **know** |
| --- | --- | --- | --- | --- | --- | --- | --- | --- | --- |
| a | Pregnant women are in general well informed about the purpose of a ***dating scan*** | □ | □ | **1** | **2** | **3** | **4** | **5** | □ |
| b | Pregnant women who undergo a ***fetal anomaly scan*** are in general well informed about the purpose and potential outcomes of the scan | □ | □ | **1** | **2** | **3** | **4** | **5** | □ |
| c | Pregnant women who undergo  ***a combined ultrasound and biochemical test (screening)*** are in general well informed about the purpose and potential outcomes of the test | □ | □ | **1** | **2** | **3** | **4** | **5** | □ |
| d | Pregnant women and health providers may have differing perspectives on the purpose of the fetal anomaly scan | □ | □ | **1** | **2** | **3** | **4** | **5** | □ |
| e | Pregnant women and health providers may have differing perspectives on the purpose of obstetric ultrasound  examinations other than the fetal anomaly scan | □ | □ | **1** | **2** | **3** | **4** | **5** | □ |
| f | Pregnant women/expectant parents expect to give birth to a healthy baby if the ultrasound does not detect any deviation | □ | □ | **1** | **2** | **3** | **4** | **5** | □ |
| g | Ultrasound findings that might indicate a deviation that is of uncertain significance often cause worry and anxiety for the pregnant woman/expectant parents | □ | □ | **1** | **2** | **3** | **4** | **5** | □ |
| h | I generally have enough time to provide counselling following an obstetric ultrasound examination when deviation(s) are detected | □ | □ | **1** | **2** | **3** | **4** | **5** | □ |

1. **Technical developments in maternity care**

| Please circle **the number** which best indicates your views about each statement | | **Strongly agree** | **Agree** | **Neutral** | **Disagree** | **Strongly disagree** |
| --- | --- | --- | --- | --- | --- | --- |
| a | Maternity care providers may trust ultrasound above clinical examinations in pregnancy | **1** | **2** | **3** | **4** | **5** |
| b | Increasing use of obstetric ultrasound may result in less focus on clinical skills | **1** | **2** | **3** | **4** | **5** |
| c | The use of ultrasound has contributed to treating pregnancy as a medical problem [medicalisation of pregnancy] | **1** | **2** | **3** | **4** | **5** |
| d | The introduction of new methods for prenatal diagnosis are seldom accompanied by appropriate ethical discussion | **1** | **2** | **3** | **4** | **5** |
| e | Possibilities for prenatal diagnosis have increased the expectation that only ‘healthy children’ should be born | **1** | **2** | **3** | **4** | **5** |
| f | Availability of prenatal diagnosis has led to a reduced acceptance of disability in our society | **1** | **2** | **3** | **4** | **5** |

1. **Maternal and fetal health interests in maternity care**

| Please circle **the number** which best indicates your views about each statement | | **Strongly agree** | **Agree** | **Neutral** | **Disagree** | **Strongly disagree** |
| --- | --- | --- | --- | --- | --- | --- |
| a | Maternity care sometimes involves prioritising between maternal and fetal health interests | **1** | **2** | **3** | **4** | **5** |
| b | The delivery sometimes has to be postponed in order to improve fetal outcome, although the pregnant woman may be at risk | **1** | **2** | **3** | **4** | **5** |
| c | Maternal health interests should always be prioritised over fetal health interests in care provided | **1** | **2** | **3** | **4** | **5** |
| d | Fetal health interests are being given more weight in decision-making, the further the gestation advances | **1** | **2** | **3** | **4** | **5** |
| e | Fetal health interests are being given more consideration in care as opportunities for fetal diagnostic and treatment develop | **1** | **2** | **3** | **4** | **5** |
| f | Fetal health interests are being given more consideration because of advances in neonatal care | **1** | **2** | **3** | **4** | **5** |
| g | Fetal health interests should be better protected by law | **1** | **2** | **3** | **4** | **5** |

1. **Decision-making in maternity care**

| Please circle **the number** which best indicates your views about each statement | | **Strongly agree** | **Agree** | **Neutral** | **Disagree** | **Strongly disagree** |
| --- | --- | --- | --- | --- | --- | --- |
| a | The final decision about receiving fetal treatment should always rest with the pregnant  woman | **1** | **2** | **3** | **4** | **5** |
| b | The decision about treatment of the fetus in utero is a shared decision between the doctor  and the pregnant woman | **1** | **2** | **3** | **4** | **5** |
| c | It is up to the doctor to decide, not the  pregnant woman, whether a fetus should be treated in utero | **1** | **2** | **3** | **4** | **5** |
| d | The expectant father has an important role in deciding whether the pregnant woman should receive treatment for the benefit of the fetus | **1** | **2** | **3** | **4** | **5** |
| e | The pregnant woman’s family (relatives other than the expectant father) has an important role in deciding whether the woman should  receive treatment for the benefit of the fetus | **1** | **2** | **3** | **4** | **5** |
| f | A pregnant woman always has the right to decline an obstetric ultrasound examination | **1** | **2** | **3** | **4** | **5** |
| g | To prevent adverse fetal outcomes, the maternity care team sometimes need to give lower priority to the health interests of the  pregnant woman | **1** | **2** | **3** | **4** | **5** |

1. **Views of the embryo and fetus**

| Please circle **the number** which best indicates your views about each statement | | **Strongly agree** | **Agree** | **Neutral** | **Disagree** | **Strongly disagree** |
| --- | --- | --- | --- | --- | --- | --- |
| a | The fetus is a *person* from the time of  conception | **1** | **2** | **3** | **4** | **5** |
| b | The fetus is a *person* from the time  heartbeats are detected | **1** | **2** | **3** | **4** | **5** |
| c | The fetus is a *person* from the time the pregnant woman experiences fetal movements | **1** | **2** | **3** | **4** | **5** |
| d | The fetus is a *person* when it can survive  outside the uterus | **1** | **2** | **3** | **4** | **5** |
| e | The fetus is a *person* when the pregnant  woman considers it to be a person | **1** | **2** | **3** | **4** | **5** |
| f | The fetus is **not** a *person* until it is born | **1** | **2** | **3** | **4** | **5** |
| g | Seeing the fetus through ultrasound makes me think of the fetus more as a  person | **1** | **2** | **3** | **4** | **5** |
| h | The fetus is a *patient* when the woman  seeks health care for her pregnancy | **1** | **2** | **3** | **4** | **5** |
| i | The fetus becomes a *patient* when fetal  abnormalities are detected | **1** | **2** | **3** | **4** | **5** |
| j | The fetus becomes a *patient* when the pregnant woman receives medical care to  enhance fetal outcome(s) | **1** | **2** | **3** | **4** | **5** |
| k | The fetus is never a patient, only the  pregnant woman can be the patient | **1** | **2** | **3** | **4** | **5** |
| l | It is the pregnant woman’s right to obtain  information about the sex of the fetus  during an ultrasound examination | **1** | **2** | **3** | **4** | **5** |
| m | There is a preference for male offspring in  my country | **1** | **2** | **3** | **4** | **5** |
| n | Sex selection is an issue in my country | **1** | **2** | **3** | **4** | **5** |

| **28.** | Do midwives perform ultrasound  at your workplace? | - Yes - No - Don’t know |
| --- | --- | --- |
| **29.** | In your view, how many ultrasound examinations are  medically indicated in an uncomplicated pregnancy? | Number of ultrasounds: |

**30. How often do you inform pregnant women about the following topics when a routine ultrasound examination is performed?**

|  |  | **Never** | **Someti mes** | **Often** | **Always** | **Not**  **relevant in my**  **setting** |
| --- | --- | --- | --- | --- | --- | --- |
| a | Purpose of the ultrasound examination | □ | □ | □ | □ | □ |
| b | The ultrasound examination is  voluntary | □ | □ | □ | □ | □ |
| c | The possibility of detection of fetal  malformation | □ | □ | □ | □ | □ |
| d | The possibility that the findings might indicate a deviation that is of uncertain  significance | □ | □ | □ | □ | □ |
| e | That the health of the fetus cannot be guaranteed even if nothing abnormal is  found | □ | □ | □ | □ | □ |
| f | Sufficient information regarding the ultrasound examination is assumed to be provided *prior* to the appointment for the examination. | - Yes - No |  |  |  |  |

**Thank you**

for completing this questionnaire about the use of ultrasound in pregnancy.

If there is anything important we have not asked about that you would like to mention, please feel free to add any comments here:
